# Extracellular vesicle features are associated with COVID‐19 severity

**DOI:** 10.1111/jcmm.17996

**Published:** 2023-11-15

**Authors:** Federica Caponnetto, Maria De Martino, Daniele Stefanizzi, Riccardo Del Sal, Ivana Manini, Feras Kharrat, Federica D'Aurizio, Martina Fabris, Daniela Visentini, Donatella Poz, Emanuela Sozio, Carlo Tascini, Daniela Cesselli, Miriam Isola, Antonio Paolo Beltrami, Francesco Curcio

**Affiliations:** ^1^ Department of Medicine University of Udine Udine Italy; ^2^ Azienda Sanitaria Universitaria Friuli Centrale Udine Italy

**Keywords:** apoptotic bodies, biomarker, COVID‐19, exosomes, extracellular vesicles, flow cytometry, machine learning, microvesicles, outcomes, SARS‐CoV2

## Abstract

COVID‐19 is heterogeneous; therefore, it is crucial to identify early biomarkers for adverse outcomes. Extracellular vesicles (EV) are involved in the pathophysiology of COVID‐19 and have both negative and positive effects. The objective of this study was to identify the potential role of EV in the prognostic stratification of COVID‐19 patients. A total of 146 patients with severe or critical COVID‐19 were enrolled. Demographic and comorbidity characteristics were collected, together with routine haematology, blood chemistry and lymphocyte subpopulation data. Flow cytometric characterization of the dimensional and antigenic properties of COVID‐19 patients' plasma EVs was conducted. Elastic net logistic regression with cross‐validation was employed to identify the best model for classifying critically ill patients. Features of smaller EVs (i.e. the fraction of EVs smaller than 200 nm expressing either cluster of differentiation [CD] 31, CD 140b or CD 42b), albuminemia and the percentage of monocytes expressing human leukocyte antigen DR (HLA‐DR) were associated with a better outcome. Conversely, the proportion of larger EVs expressing N‐cadherin, CD 34, CD 56, CD31 or CD 45, interleukin 6, red cell width distribution (RDW), N‐terminal pro‐brain natriuretic peptide (NT‐proBNP), age, procalcitonin, Charlson Comorbidity Index and pro‐adrenomedullin were associated with disease severity. Therefore, the simultaneous assessment of EV dimensions and their antigenic properties complements laboratory workup and helps in patient stratification.

## 1 INTRODUCTION

Following the first retrospectively identified cases of severe acute respiratory syndrome‐coronavirus 2 (SARS‐CoV2) infection in humans, dating back to December 2019,^1^ the novel coronavirus infection spread in a tumultuous fashion, and as early as March 2020, the World Health Organization (WHO) declared the coronavirus‐induced disease (COVID‐19) a pandemic.^2^ Only 1 month after the severe acute respiratory syndrome‐coronavirus 2 (SARS‐CoV2) outburst in Wuhan, two cases of COVID‐19 were first reported in Italy, and the nation was in lockdown as soon as March 2020.^3^ Thanks to enormous scientific effort, at the end of 2020, the vaccination campaign started in Italy, and by the end of 2021, 108 million doses were administered, so that 88.7% of people older than 12 years had received at least one dose.^4^ Although the vaccine shows less protection against infection, it dramatically reduces the number of severe COVID‐19 cases.^5^ However, the protection seems to wane over time.^5^ To add a layer of complexity, due to the high mutation rates of this RNA virus, different strains have emerged over the years, which were characterized by higher infectivity and either higher or lower pathogenicity with respect to the Wuhan strain.^6^ The emergence of new variants with the potential to evade the immune system poses a risk to the recrudescence of pandemics.

From a clinical point of view, there is a need to identify early biomarkers of adverse outcomes that could help clinicians rapidly discern, among patients with SARS‐CoV2 infection and pneumonia, those that will evolve into a critical disease or succumb to COVID‐19. For this purpose, we have already employed targeted^7^ or high‐throughput serum proteomics analyses,^8^ demonstrating that inflammatory markers, including interleukin‐6, are among the strongest predictors of patient outcome, even when analysed in combination with routine haematological, blood chemistry, demographic and clinical data of enrolled patients.^8^

Extracellular vesicles (EV) are cell fragments enclosed in the plasma membrane that are produced in response to a variety of physiological and pathological stimuli. EV can be divided into three broad subgroups (apoptotic bodies, microvesicles and exosomes) based on their size, which partly reflects their mechanism of origin.^9^, ^10^ EV can reflect ongoing pathological processes, such as apoptosis and viral infection,^10^, ^11^ but also play a very important role in physiological processes, ranging from coagulation to cell–cell communication, and regulation of inflammation and immune responses.^9^, ^12^ An accumulating body of literature has demonstrated the potential involvement of EV in COVID‐19 pathophysiology. Therefore, we assessed whether EV characteristics could be used as biomarkers for a critical disease.

For this purpose, we enrolled 146 patients who were affected by either severe or critical disease and analysed blood samples collected within 1 day (median) of hospital admission. Cohort data regarding demographic characteristics, comorbidities, routine haematology, blood chemistry, flow cytometric analysis of lymphocyte subpopulations and EV characterization were collected and analysed using machine learning algorithms. Elastic net logistic regression analysis with cross‐validation showed that features of smaller EVs (i.e. the fraction of EVs smaller than 200 nm expressing either CD 31, CD 140b or CD 42b) were predictors of a better outcome, together with albuminemia and percentage of monocytes expressing human leukocyte antigen DR (HLA‐DR). Conversely, the proportion of large EVs expressing either N‐cadherin, CD 34, CD 56, CD 31 or CD 45 was associated with disease severity, together with interleukin 6, red cell width distribution (RDW), N‐terminal pro‐brain natriuretic peptide (NT‐proBNP), age, procalcitonin, Charlson Comorbidity Index and pro‐adrenomedullin. Taken together, our data suggest that EV may be employed as biomarkers for critical COVID‐19. Furthermore, subsets of small EVs may promote a less aggressive outcome in SARS‐CoV2 infection.

## 2 MATERIALS AND METHODS

### 2.1 Enrolled patients and clinical data

This study was authorized by the Regional Ethics Committee (2020‐Os‐033; Em. Sost. N. 1 versione 1 data, 16.08.2021) and conducted according to the declaration of Helsinki, and signed informed consent was collected from enrolled patients and controls. The inclusion criteria for patients were age >18 years and nasopharyngeal swab positivity for the SARS‐CoV‐2 genome.

We classified ‘critical’ and ‘severe’ patients according to the revised 2023 WHO clinical management assification.^13^ Relevant clinical, demographic, haematological, blood chemistry and flow cytometric data were collected from hospital electronic health records (INSIEL) and pseudonymized.

### 2.2 Sample collection and preservation

Blood samples were drawn within 1 day (median value) of hospital admission and collected into 5‐mL EDTA tube for plasma separation (Vacuette, Greiner Bio‐One). Platelet‐poor plasma (PPP) is obtained by centrifuging blood at 1400 × *g* for 15 min, followed by centrifugation at 14,000 × *g* for 20 min at +4°C for platelet depletion. The plasma was immediately stored at −80°C until use. Aliquots of PPP samples obtained from 10 patients were analysed using a clinical haemocytometer (Coulter) to assess the levels of platelet contamination.

### 2.3 Extracellular vesicles isolation and analysis

To isolate small extracellular vesicles (EV) from PPP, we employed the ExoQuick Exosome Precipitation Solution (System Biosciences) following the protocol described in reference.^14^ To remove larger EV, we filtered them with a 0.2‐μm syringe filter following isolation. Nanoparticle tracking analysis was conducted employing Nanosight (LM10, Malvern System Ltd., London, UK), equipped with a 405‐nm laser, as in reference.^14^

### 2.4 Flow cytometry analysis

To identify EV, we stained the cytoplasm with carboxyfluorescein succinimidyl ester (CFSE; Invitrogen). Directly conjugated antibodies against CD31 (Miltenyi Biotec), CD45 (Dako), N‐cadherin, CD56, CD140b, CD34 and CD42b (BD Biosciences) were used to characterize EV subpopulations. CD63 was used to assess whether exosomes fell below the limit of detection in our system.

Briefly, 15‐mL PPP was incubated with 1:1 CFSE (40 mM in DMSO) for 2 h at 37°C. Then, 1.2 mL of each antibody was incubated for 30 min at +4°C. DMSO and isotype‐matched antibodies were used as the negative controls.

**FIGURE 1:**
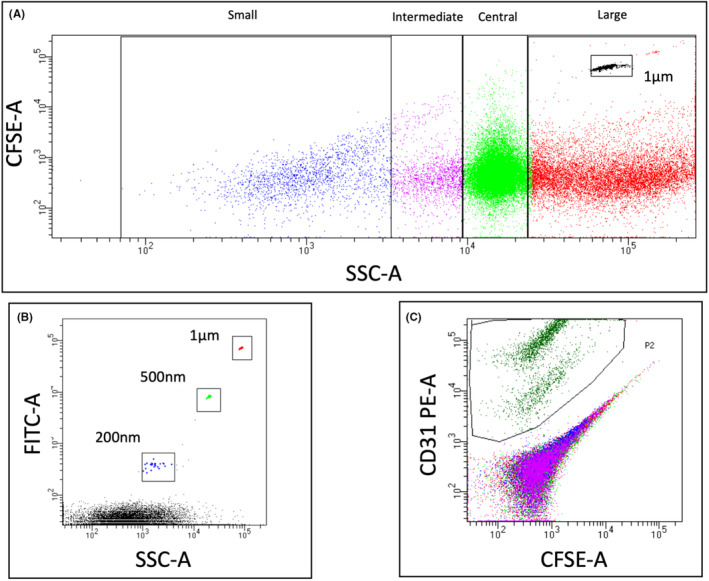
Gating strategy employed to analyse EV. A gating strategy was adopted to analyse the plasma EVs of the enrolled patients. (A) CFSE staining was used to identify EV cytoplasm. 1‐μm‐size particles were added to the CFSE‐stained EV to quantify their concentration. (B) The EV size was determined based on SSC‐A. Fluorescent particles of known size were used to calibrate the instrument. (C) CFSE‐labelled EV were stained with antibodies specific to the antigens of interest to determine the immunophenotype of each EV subset. A typical example of a CD31‐ and CFSE‐labelled plasma sample is shown.

The samples were analysed using a FACSCanto II flow cytometer (Becton Dickinson). EV sizes were estimated using side scatter (SSC), while flow cytometry sub‐micron size (200–500–1000 nm) reference beads (Invitrogen) were employed to calibrate the instrument and quantify vesicle concentration. A minimum of 50,000 events were recorded per sample. Data were analysed using BDFACSDiva Software (Becton Dickinson). The gating strategy is illustrated in Figure 1.

Based on EV size, we identified four subpopulations at SSC (i.e. small ≤200 nm, intermediate ≈200–400 nm, central ≈500 nm and large >500 nm). For our analysis, we computed: (a) The proportion of EV included in the four SSC subpopulations, named small EV (%), intermediate EV (%), central EV (%) and large EV (%); (b) the proportion of the whole EV population expressing one of the above‐mentioned surface markers, named CD31^+^ EV (%), CD34^+^ EV (%), CD42b^+^ EV (%), CD45^+^ EV (%), CD140b^+^ EV (%), CD56^+^ EV (%) and N‐cadherin^+^ EV (%); and (c) the proportion of each SSC subpopulation of EV expressing one of the above‐mentioned surface markers, named small EV CD31^+^ (%), intermediate EV CD31 CD31^+^ (%), central EV CD31^+^ (%) and large CD31^+^ (%). By adding a specified number of reference beads to the PPP samples, we also computed the EV concentration.

### 2.5 Statistics

Descriptive statistics for categorical variables are presented as number (percentage), and continuous variables as mean ± standard deviation (SD) or median (interquartile range [IQR]). Normality was assessed using the Shapiro–Wilk test. Comparisons between categorical variables were performed using the chi‐square test or Fisher's exact test, as appropriate. Comparisons between continuous variables were performed using the *t*‐test or Mann–Whitney *U*‐test, as appropriate, and corrected for multiple testing using the False Discovery Rate correction method. An elastic net logistic regression algorithm was used to build different models to predict ‘critical’ or ‘severe’ outcomes. The elastic net logistic regression is a regularization model that combines, through a linear combination of LASSO and Ridge methods, both L1 and L2 penalties. This model performs variable selection, forming a subset of predictors, each of which is matched with a regression coefficient. Variable importance was ordered using the absolute value of the regression coefficient; a higher value indicated a larger contribution to the model. The dataset was split into testing (30%) and training (70%) sets. A 10‐fold cross‐validation was applied to the training set to tune the hyperparameters λ, determine the amount of shrinkage and α and explain the presence of L1 and L2 penalties. The model was trained separately for the demographic, comorbidity, haematological, blood chemistry, EV and PBMNC data. Furthermore, all the variables of the training set with a corrected *p* value < 0.05 were included in the final model. Finally, we added the following clinically relevant confounders to the complete model: lymphocyte relative counts, together with the fraction of T CD4^+^ helper lymphocytes and activated CD14^+^ HLA‐DR^+^ monocytes. The performance of these models was evaluated on the testing set using the receiver operating curve (ROC) and the area under the curve (AUC), with a 95% confidence interval (CI), and compared using Long's test. Correlations between the variables selected by the final model were investigated using a correlation heatmap with the Spearman method. Analyses were performed using STATA version 17.

## 3 RESULTS

### 3.1 Demographic and clinical characteristics of enrolled patients

A total of 146 patients were enrolled between November 2020 and April 2021, and B.1.177 and Alpha (B.1.1.7) variants were the most prevalent genotypes in Italy.^15^ All enrolled subjects were positive for SARS‐CoV‐2, and samples were collected within a median of 1 day after hospital admission. At the time of enrollment, no patient had completed the vaccination scheme. According to the 2022 guidelines for COVID‐19 care, patients were dichotomized as severe, which either had oxygen saturation <90% in room air or showed signs of pneumonia or severe respiratory syndrome, and critical, which required life‐sustaining treatment or displayed acute respiratory distress syndrome, sepsis or death.^13^

Table 1 shows the comorbidity scores and the demographic data of the enrolled patients. The patients were mainly male, older than 70 years, with a median Charlson Comorbidity Index (CCI) of 4. Critical patients were significantly older and had a significantly higher CCI than those with severe COVID‐19. Consistent with the inclusion criteria, all patients who died were critical.

**TABLE 1:** Demographic and clinical features of enrolled patients.

	Total (*N* = 146)	Severe (*N* = 83)	Critical (*N* = 63)	*p* Value
Charlson comorbidity index, median (IQR)	**4 (3–6)**	**3 (2–6)**	**5 (4–7)**	**<0.001**
Age, years, median (IQR)	**73 (65–80)**	**71 (57–78)**	**77 (69–81)**	**0.002**
Sex, *n* (%)				
Female	39 (26.7)	21 (25.3)	18 (28.6)	0.658
Male	107 (73.3)	62 (74.7)	45 (71.4)	
Death (%)	**29**	**0**	**68**	**<0.001**

*Note*: Data are shown as median and interquartile range (IQR) or percentages. The *p* value refers to the comparison between the severe and critical patients. The significant results are shown in bold.

### 3.2 Haematological and blood chemistry data associated with patient outcome

Table 2 shows the haematological and blood chemistry variables of all the enrolled patients stratified according to their outcomes. The levels of two sepsis‐associated markers (pro‐adrenomedullin and procalcitonin) were significantly higher in critical patients. Similarly, the cardiac injury marker troponin‐T and heart failure marker N‐terminal pro‐B‐type natriuretic peptide were significantly higher in critical patients. Concerning haematological data, red cell width distribution and lymphocyte counts were the only two parameters that significantly differed between severe and critical patients. Finally, the levels of the inflammatory cytokine interleukin 6 were significantly increased in critically ill patients.

**TABLE 2:** Hematologic and blood chemistry data of the enrolled patients.

Median (IQR)	Total (*N* = 146)	Severe (*N* = 83)	Critical (*N* = 63)	*p* Value	Corrected *p* value
C reactive protein (mg/L)	85.31 (48.7–129.4)	77.2 (43.4–121.8)	102.5 (50.9–142.1)	0.031	0.083
Pro‐adrenomedullin (nmol/L)	**1.14 (0.9–1.7)**	**1.1 (0.8–1.3)**	**1.4 (1–2.0)**	**<0.001**	**0.014**
Procalcitonin (ng/mL)	**0.13 (0.1–0.3)**	**0.11 (0.06–0.20)**	**0.19 (0.09–0.46)**	**<0.001**	**0.028**
Albumin (g/L)	33.5 (31–36)	34 (32–37)	32 (29–36)	0.005	0.056
Total bilirubin (mg/dL)	0.51 (0.4–0.7)	0.48 (0.40–0.67)	0.60 (0.39–0.78)	0.178	0.146
Blood urea nitrogen (mg/dL)	24.5 (18–33)	23 (17–30)	27 (21–37)	0.012	0.063
Chloride (mmol/L)	100 (98–102.5)	100 (98–102)	101 (97–103)	0.500	0.181
Creatine phosphokinase (U/L)	79 (41–195)	70 (37–134)	105 (49–254)	0.128	0.125
Creatinine (mg/L)	1.04 (0.9–1.2)	1.01 (0.87–1.25)	1.04 (0.87–1.27)	0.536	0.194
Glucose (mg/dL)	125 (102.5–163.5)	126 (103–158)	125 (100–167)	0.971	0.236
Aspartate amino transferase (U/L)	32.5 (22.5–44)	30 (22–42)	38 (23–44)	0.069	0.111
Alanine amino transferase (U/L)	26 (16–46)	25 (16–46)	34 (16–49)	0.490	0.174
Potassium (mEq/L)	4.25 (3.9–4.5)	4.2 (3.9–4.6)	4.3 (3.9–4.5)	0.950	0.229
Lactate dehydrogenase (U/L)	719 (551–945)	680 (499–786)	841 (621–992)	0.139	0.132
Sodium (nmol/L)	139 (137–142)	139 (137–141)	140 (127–142)	0.163	0.139
Plasmatic osmolarity (mOsm/L)	285 (279–290)	284 (279–288)	287 (280–293)	0.051	0.104
N‐terminal pro‐B‐type natriuretic peptide (pg/mL)	**686 (233–1979)**	**461 (192–1175)**	**982 (451–3548)**	**<0.001**	**0.020**
Troponin‐T (μg/L)	**17.7 (8.8–44.3)**	**11.8 (7.3–29.8)**	**28.4 (15–67.4)**	**<0.001**	**0.007**
White blood cells (10^3^/μL)	7.55 (5.5–10.2)	7.4 (5.4–10.0)	7.7 (5.6–11.5)	0.411	0.160
Red blood cells (10^6^/μL)	4.38 (4–4.7)	4.4 (4.0–4.7)	4.4 (3.7–4.7)	0.823	0.208
Haemoglobin (g/dL)	13.2 (12–14.2)	13.1 (12.1–14)	13.4 (11.6–14.2)	0.933	0.222
Haematocrit (%)	39.3 (35.3–42.1)	38.8 (35.9–41.9)	40.5 (34.1–42.4)	0.995	0.250
Mean corpuscular volume (fL)	90.6 (87.6–94.6)	91 (88–94.6)	90.6 (87.2–94.9)	0.822	0.201
Mean corpuscular haemoglobin (pg)	30.6 (29.4–31.9)	30.6 (29.5–31.9)	30.7 (29.2–31.9)	0.861	0.215
Mean corpuscular haemoglobin concentration (%)	33.6 (33.1–34.1)	33.6 (33–34.1)	33.5 (33.1–34.2)	0.976	0.243
Red cell distribution width (%)	**13.7 (13.2–15.2)**	**13.5 (13.1–14.6)**	**14.1 (13.5–16)**	**0.003**	**0.049**
Platelets (10^3^/μL)	228 (170–304)	247 (186–306)	189 (146–292)	0.013	0.076
Neutrophils (%)	85.6 (80.3–90.7)	84.1 (79.9–89.2)	88.2 (81.9–91.9)	0.012	0.069
Lymphocytes (%)	**7.7 (4.7–11.8)**	**8.9 (6.1–12)**	**5.9 (4–9.8)**	**0.002**	**0.042**
Monocytes (%)	5.2 (3.8–8)	5.5 (4.1–8.4)	4.7 (3–7.4)	0.039	0.090
Eosinophils (%)	0 (0–0.1)	0 (0–0.2)	0 (0–0.1)	0.043	0.097
Basophils (%)	0.2 (0.1–0.3)	0.2 (0.1–0.4)	0.2 (0.1–0.3)	0.092	0.118
D‐dimer (ng/mL)	916 (590–2093)	893 (573–2093)	955 (626–1995)	0.471	0.167
Prothrombin time ratio	1.13 (1.1–1.2)	1.12 (1.07–1.23)	1.18 (1.06–1.28)	0.382	0.153
Prothrombin time international normalized ratio	1.12 (1.1–1.2)	1.11 (1.06–1.22)	1.14 (1.06–1.26)	0.533	0.188
Interleukin 6 (pg/mL)	**35.65 (19–96)**	**27.3 (11.9–48)**	**55 (22–116.7)**	**0.001**	**0.035**

*Note*: Data are shown as median and interquartile range (IQR) or percentages. The *p* value is the result of the comparison between the severe and critical patients, and the last column shows the *p* values corrected for multiple comparisons. The significant results are shown in bold.

### 3.3 Leukocyte and extracellular vesicles subpopulation data associated with patient outcome

Since COVID‐19‐critical patients are characterized by a reduced lymphocyte count and immunosuppression, we decided to include in our analysis the results of the characterization of peripheral blood mononuclear cells (PBMC), as determined by flow cytometry. As shown in Table 3, the fraction of monocytes expressing HLA‐DR was significantly lower in critical patients than in severe patients. Aside from the fraction of CD4^+^ CD3^+^ T helper lymphocytes, which had marginal significance, the other populations did not significantly differ between the two patient subsets.

**TABLE 3:** Peripheral blood mononucleated cell immunophenotypes of the enrolled patients.

Median (IQR)	Total (*N* = 146)	Severe (*N* = 83)	Critical (*N* = 63)	*p* Value	Corrected *p* value
T helper lymphocytes CD4^+^ CD3^+^ (%)	**44 (34–52)**	**44.5 (35–53)**	**42 (32–50)**	**0.212**	**0.050**
Cytotoxic/suppressor T lymphocytes CD8^+^/CD3^+^ (%)	18 (14–28)	19 (14–27)	18 (13–29)	0.794	0.225
Natural killer (NK) CD56^+^ CD16^+^ (%)/CD3^−^	17 (10–26)	16.5 (10–22)	19 (10–29)	0.377	0.075
B lymphocytes CD19^+^ (%)	14 (8–20)	13 (8.5–19)	15 (8–20)	0.775	0.200
NK‐like T lymphocytes CD3^+^/CD56 CD16^+^ (%)	5 (2.2–9)	5 (2.4–9.2)	5 (1.3–9)	0.377	0.100
Activated T lymphocytes CD3^+^ HLA‐DR^+^ (%)	12 (8–16)	12 (8–16)	11 (7–17)	0.687	0.175
Activated T helper lymphocytes CD3^+^ CD4^+^ HLA‐DR^+^ (%)	5 (3–7)	5 (3–7)	5 (3–7)	0.603	0.150
Activated T cytotoxic lymphocytes CD3^+^ CD8^+^ HLA‐DR^+^ (%)	6 (4–9)	7 (4–8)	6 (4–9)	0.975	0.250
Recent thymic emigrants (RTE; %)	17.1 (10.1–25.8)	18.4 (11.1–27.8)	16.4 (10.1–24)	0.589	0.125
Monocytes HLA‐DR^+^ (%)	**98 (91.6–99.3)**	**98.3 (96–99.5)**	**97 (87–99.1)**	**0.037**	**0.025**

*Note*: Data are shown as median and interquartile range (IQR) or percentages. The *p* value is the result of the comparison between the severe and critical patients, and the last column shows the *p* values corrected for multiple comparisons. The significant results are shown in bold.

To identify novel markers associated with COVID‐19 outcomes, we characterized the surface phenotype of circulating EVs of micro‐ and nanometre dimensions. Platelet‐poor plasma (estimated level of platelet contamination: 3.88 ± 1.82·10^3^ particles/μL, *n* = 10) was analysed by flow cytometry to quantify EVs, assess their size (dividing them into small ≤200 nm, intermediate ≈200–400 nm, central ≈500 nm and large >500 nm subgroups) and analyse the expression of markers of endothelial, platelet, leukocyte, natural killer (NK) cells, mural cell and neural cell origin (i.e. CD31, CD34, CD42b, CD45, CD140b, CD56 and N‐cadherin). EVs were analysed according to the gating strategy shown in Figure 1.

As shown in Table 4, the fraction of EV with small dimensions is significantly, although marginally, higher in severe patients than in critical patients. Similarly, the fraction of small vesicles expressing CD31, CD42b or CD140b was significantly lower in the plasma of critical patients. Conversely, the fraction of EV of ≈500‐nm diameter that expressed CD140b or CD56 and the fraction of EV of >500‐nm diameter that expressed either CD34 or CD45 showed an opposite trend, being higher in critical patients.

**TABLE 4:** Flow cytometry analysis of plasma extracellular vesicles (EVs) from the enrolled patients.

Median (IQR)	Total (*N* = 146)	Severe (*N* = 83)	Critical (*N* = 63)	*p* Value	Corrected *p* value
CD31^+^ EV (%)	1.8 (0.8–7.2)	1.8 (1–7.3)	1.8 (0.5–6)	0.467	0.194
CD34^+^ EV (%)	0.3 (0.1–0.4)	0.3 (0.1–0.4)	0.3 (0.2–0.5)	0.271	0.169
CD42b^+^ EV (%)	1 (0.4–3.3)	0.9 (0.3–2.7)	1.2 (0.5–3.7)	0.160	0.144
CD45^+^ EV (%)	0.3 (0.1–0.5)	0.3 (0.1–0.5)	0.3 (0.1–0.6)	0.892	0.238
CD140b^+^ EV (%)	1.9 (0.8–3.5)	1.9 (0.7–3.3)	2.1 (0.8–4.5)	0.231	0.156
CD56^+^ EV (%)	0.6 (0.2–1.3)	0.6 (0.2–1.6)	0.6 (0.3–1.3)	0.328	0.175
N‐cadherin^+^ EV (%)	3.1 (1.5–5.2)	3.4 (1.5–5.2)	2.8 (1.5–5.2)	0.617	0.213
EV/μL	1748.8 (972.4–3921. 7)	1797.7 (999.0–4318.6)	1604.4 (952.9–3241.4)	0.603	0.206
Small EV (%)	**20.6 (10.2–31.8)**	**22.8 (10.5–37.3)**	**14.6 (8.5–23.9)**	**0.030**	**0.050**
Intermediate EV (%)	10.9 (6.7–20.5)	11.6 (7.3–21.0)	9.4 (6.7–20.5)	0.381	0.181
Central EV (%)	31.5 (21.1–46.3)	28.9 (18.4–43.1)	36.9 (24.1–49.9)	0.030	0.056
Large EV (%)	21.35 (12.9–36.5)	19.8 (12.8–32.9)	22.8 (13.2–41.4)	0.454	0.188
Small EV CD31^+^ (%)	**5.18 (1.9–10.2)**	**7.5 (3.0–13.3)**	**3.2 (1.1–6.9)**	**<0.001**	**0.012**
Intermediate EV CD31^+^ (%)	28.1 (16–41.6)	30.2 (16.9–45)	25.3 (13.1–36.2)	0.071	0.088
Central EV CD31^+^ (%)	33.26 (23.7–44.2)	31 (23.1–42.0)	35 (28.0–47.3)	0.138	0.119
Large EV CD31^+^ (%)	23.64 (14–47.4)	23.2 (11.1–41.7)	25.0 (16–50)	0.158	0.138
Small EV CD34^+^ (%)	2.90 (0–7.42)	3.9 (0–8.9)	2.4 (0–5.0)	0.149	0.131
Intermediate EV CD34^+^ (%)	4.52 (1.4–10.2)	5.3 (0.8–12.5)	4.1 (1.7–8.3)	0.222	0.150
Central EV CD34^+^ (%)	13 (6.8–19)	13.3 (8.2–19.7)	11.1 (5.4–18.4)	0.101	0.100
Large EV CD34^+^ (%)	**77.91 (64.3–86.8)**	**72.9 (54.5–84.4)**	**80.2 (72.1–89.2)**	**0.002**	**0.037**
Small EV CD42b^+^ (%)	**6.14 (2.4–10.2)**	**8.3 (3.7–13.6)**	**4.1 (1.9–7.1)**	**<0.001**	**0.019**
Intermediate EV CD42b^+^ (%)	26.1 (16.4–35.1)	27.6 (19.3–35.5)	20.4 (12.3–33.9)	0.143	0.125
Central EV CD42b^+^ (%)	36.02 (23.1–46.4)	33.9 (24.0–45.2)	36.3 (21.8–49.0)	0.776	0.231
Large EV CD42b^+^ (%)	25.2 (14.2–43.5)	23.0 (13.6–37.2)	30.4 (14.5–60.6)	0.031	0.063
Small EV CD45^+^ (%)	1.07 (0–2.7)	1.5 (0–3.2)	0.2 (0–2.3)	0.069	0.081
Intermediate EV CD45^+^ (%)	2.65 (0–5.2)	3.2 (0–7.1)	2 (0–4.3)	0.115	0.106
Central EV CD45^+^ (%)	8.25 (4.1–13.2)	8.9 (5.6–13.6)	7.1 (3.2–11.3)	0.118	0.113
Large EV CD45^+^ (%)	**87.74 (80–93.4)**	**84.1 (72.7–91.2)**	**90.3 (83.9–94.5)**	**<0.001**	**0.025**
Small EV CD140b^+^ (%)	**2.3 (0.7–4)**	**2.4 (1.5–4.6)**	**1.5 (0.4–2.7)**	**<0.001**	**0.031**
Intermediate EV CD140b^+^ (%)	7.5 (4.8–10)	8.1 (5.4–10.8)	6.4 (4.5–8.8)	0.032	0.069
Central EV CD140b^+^ (%)	28.32 (22.8–33.5)	**25.2 (20.4–31.1)**	**30.4 (25.5–34.7)**	**<0.001**	**0.006**
Large EV CD140b^+^ (%)	63.41 (56–69.3)	63.2 (54.0–70.4)	63.7 (57.9–69.1)	0.598	0.200
Small EV CD56^+^ (%)	2.1 (0.6–5.2)	2.1 (0.3–5.5)	2 (0.9–5.0)	0.921	0.244
Intermediate EV CD56^+^ (%)	8.14 (3.9–13.7)	7.6 (3.1–14.1)	9.2 (5.0–13.3)	0.244	0.163
Central EV CD56^+^ (%)	**24.15 (16.9–28.8)**	**23.2 (15.8–28.3)**	**25.3 (21.3–29.6)**	**0.015**	**0.044**
Large EV CD56^+^ (%)	61.35 (52.5–70)	60 (46.5–68.2)	65.1 (55.7–70.6)	0.085	0.094
Small EV N‐cadherin^+^ (%)	7 (3.5–13.9)	7 (2.8–18.9)	7 (3.9–13.1)	0.732	0.225
Intermediate EV N‐cadherin^+^ (%)	5.34 (3.1–9.7)	5.2 (3.1–9.9)	5.4 (3.1–8.7)	0.964	0.250
Central EV N‐cadherin^+^ (%)	16.14 (10.8–24.1)	14.8 (10.3–23.2)	19.7 (11.9–28.0)	0.038	0.075
Large EV N‐cadherin^+^ (%)	67.33 (47.3–79.7)	65.9 (36.2–80.2)	67.5 (50.3–78.9)	0.668	0.219

*Note*: Data are shown as median and interquartile range (IQR) or percentages. The *p* value is the result of the comparison between the severe and critical patients, and the last column shows the *p* values corrected for multiple comparisons. The significant results are shown in bold.

Importantly, CD63 expressing EV could not be detected using our setup, suggesting that exosomes fell under the limit of detection of our system (Figure S1).

### 3.4 Identification of independent predictors of patient outcome

Next, we attempted to identify, employing an elastic net logistic regression analysis with cross‐validation, the variables needed to build the best model to classify critical patients. The area under the curve (AUC) of the receiver operator curve (ROC) was used to evaluate model performance. Three models were tested. To build the first model, we employed all demographic, comorbidity, haematological and blood chemistry data. Table 5 lists the variables and coefficients used to build the model. ROC AUC was 0.750 (95% CI 0.607–0.892).

**TABLE 5:** Results of the elastic net logistic regression analysis of demographic, comorbidity, haematological and blood chemistry data of the enrolled patients.

Variable	Coefficient
Interleukin 6	0.301
Age	0.185
Lactate dehydrogenase	0.153
Charlson Comorbidity Index	0.130
Red cell width distribution	0.126
Albumin	−0.117
Platelets	−0.111
Pro‐adrenomedullin	0.081
NT‐pro‐brain natriuretic peptide	0.062
Lymphocytes	−0.062
Procalcitonin	0.056
Creatin phosphokinase	−0.055
Prothrombin time (INR)	−0.053

*Note*: The variables included in the model are listed, along with their coefficients.

To build the second model, we employed all the EV data. Table 6 lists the variables and coefficients used to construct the model. ROC AUC was 0.663 (95% CI 0.507–0.819).

**TABLE 6:** Results of the elastic net logistic regression analysis of the extracellular vesicles (EV) analysis data of the enrolled patients.

Variable	Coefficient
Small EV CD31^+^	−0.496
Intermediate EV NCAD^+^	0.414
Large EV CD56^+^	0.207
Small EV CD140b^+^	−0.193
Large EV CD34^+^	0.189
Central EV CD56^+^	0.178
Large EV CD45^+^	0.159
Large EV CD42b^+^	0.139
Small EV CD45^+^	−0.120
NCAD^+^ EV	−0.119
Small EV CD56^+^	−0.082
Small EV CD42b^+^	−0.060
CD140b^+^ EV	0.55
Central EV CD31^+^	0.046
CD56^+^ EV	−0.014
Central EV CD34^+^	−0.004
Central EV NCAD^+^	0.001

*Note*: The variables included in the model are listed, along with their coefficients.

Next, we built a model that included all the PBMC immunophenotype data. The model did not discriminate the outcome, being the ROC AUC 0.520 (95% CI 0.350–0.690).

**FIGURE 2:**
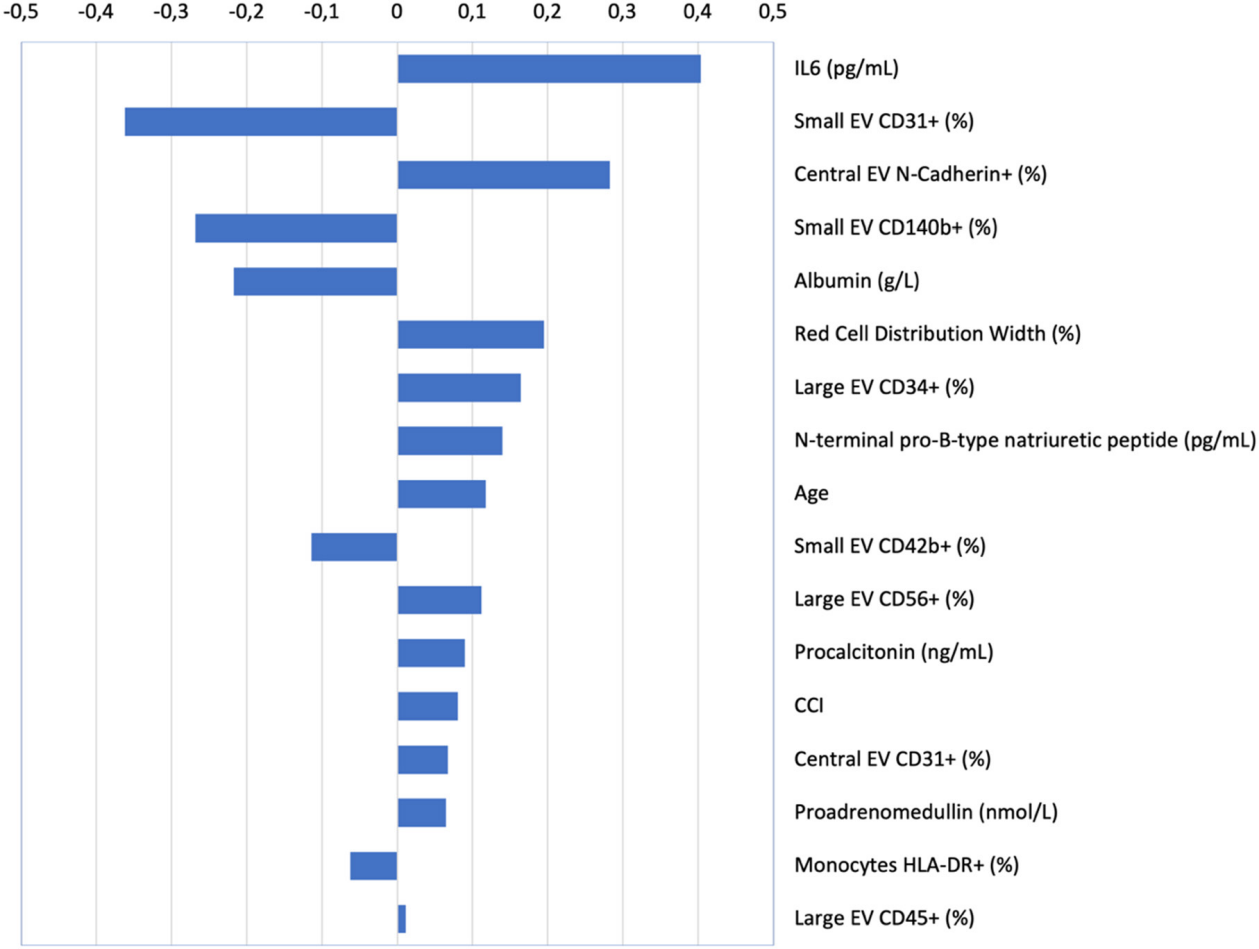
Results of the elastic net logistic regression analysis of the complete model. Results of the elastic net logistic regression model comprising all the significant variables. Each variable included in the model is a row on the *y*‐axis (labels are shown on the right side of the panel), while the coefficients are plotted as bars on the *x*‐axis.

Next, we built a complete model, including demographic, comorbidity, haematological, blood chemistry, EV and PBMNC data with a corrected *p* value < 0.05 in the training set (Tables S1–S4). The results are shown in Figure 2. ROC AUC was 0.769 (95% CI 0.6030–0.908). Even when we added to the complete model, other potential confounding factors (i.e. lymphocyte relative counts, the fraction of T CD4^+^ helper lymphocytes and activated CD14^+^ HLA‐DR^+^ monocytes), the fraction of small vesicles expressing CD31 and the fraction of central EV expressing N‐cadherin remained among the strongest predictors of patient outcome (Figure S2), while the ROC AUC increased marginally; 0.785 (95% CI 0.650–0.919).

**FIGURE 3:**
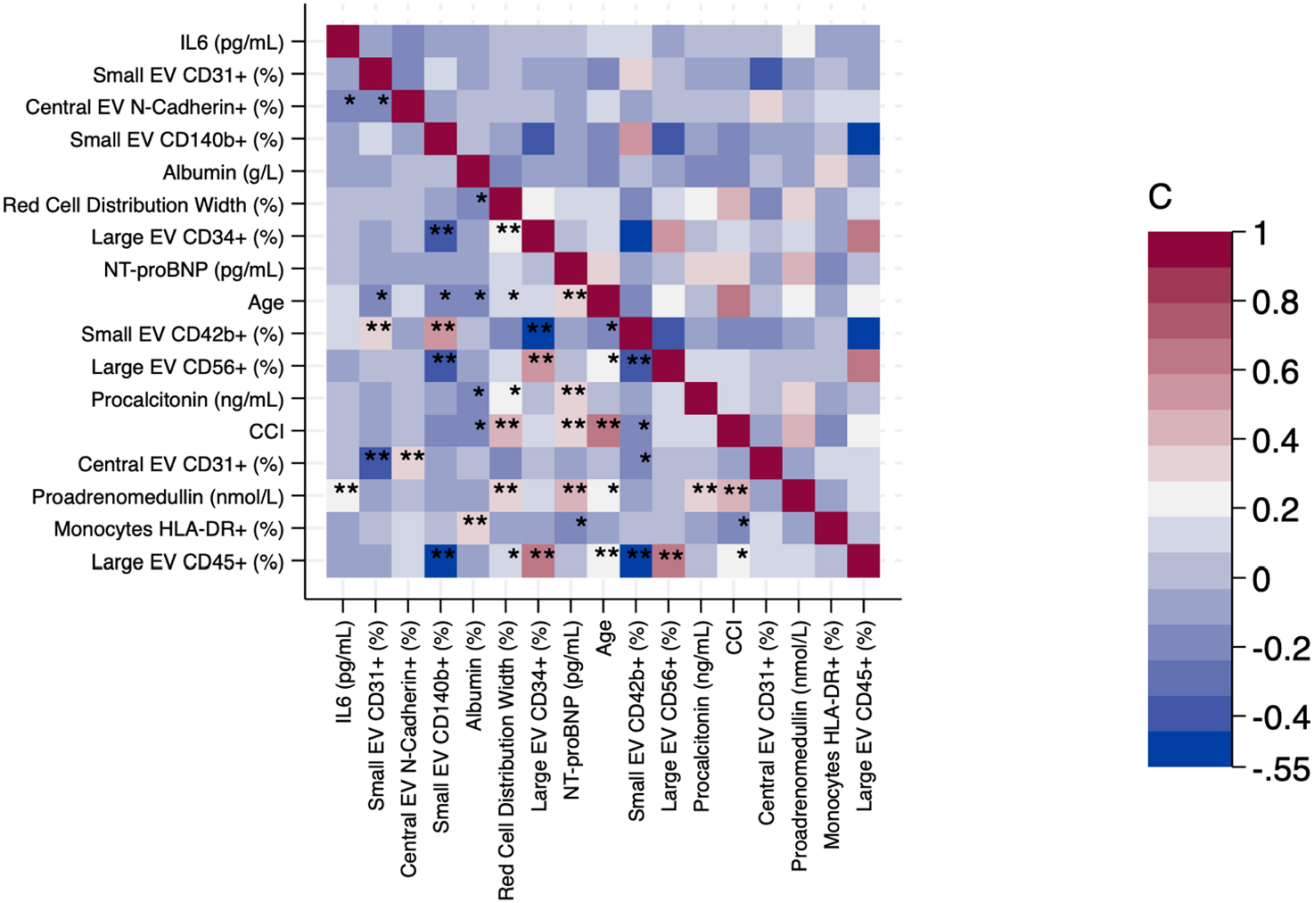
Correlation among the variables of the complete model. Heatplot summarizing the results of the correlation analysis between variables emerging from elastic net logistic regression. Correlation coefficients are shown as a heatmap, according to the legend on the right side of the panel. *p < 0.05, **p < 0.01.

Finally, we analysed the correlations among the variables of the complete model (Figure 3).

Although most of the vesicle‐related parameters correlated with each other, interleukin 6 showed a negative correlation with the fraction of ≈500‐nm‐diameter EVs expressing N‐cadherin, while age was directly correlated with the fraction of large EV expressing CD45 or CD56 and inversely correlated with the fraction of small EV expressing CD31, CD42b or CD140b. In line with this, the Charlson Comorbidity Index was directly correlated with the fraction of large EV expressing CD45 and inversely correlated with the fraction of small EV expressing CD42b. Intriguingly, RDW also correlated with the fraction of large EV expressing either CD34 or CD45.

## 4 DISCUSSION

In this study, we present data concerning the extensive characterization, from a clinical and laboratory point of view, of 146 hospitalized patients affected by SARS‐CoV‐2 infection recruited at our institution between November 2020 and April 2021, during a phase of the pandemic in which B.1.177 and Alpha (B.1.1.7) were the most prevalent genotypes in Italy.^15^ No patient in our series was fully vaccinated since the vaccination campaign began in Italy at the end of 2020, and by March 2021, only a small minority of the population had completed the entire scheme.^16^ We decided to analyse only patients with severe or critical COVID‐19 to understand the critical variables that could help clinicians identify, among hospitalized patients, those that need to be more carefully monitored.

Here, we specifically focused our attention on circulating EV, since an accumulating body of literature has shown their crucial role in both homeostatic and pathological processes, including infectious diseases, immune system regulation, coagulation and tissue repair.^9^ The term EV is broad and includes heterogeneous populations of cell‐derived membrane‐enveloped particles that differ in size, content composition, biogenesis and biological function.^9^ Three main subtypes of EV can be identified based on their size and biological functions: exosomes, microvesicles and apoptotic bodies. Exosomes are formed intracellularly within multivesicular bodies, have a small size (<200‐nm diameter) and can be released in the extracellular space, where they influence the behaviour of target cells either via ligand‐receptor interactions, or following phagocytosis, pinocytosis or by fusing the plasma membrane with the target cells, thus releasing their content into recipient cells.^10^ Microvesicles have larger dimensions (100–1000 nm in diameter) and are formed by budding of the plasma membrane. Therefore, their composition reflects, possibly more closely than that of exosomes, the features of their cell of origin.^10^ Finally, EV comprise apoptotic bodies, which have even larger dimensions (1000–5000‐nm diameter) and are formed during this form of programmed cell death.^10^ To add a layer of complexity, more recently it has been shown that, similarly to healthy cells, apoptotic cells may release microvesicles and exosomes too.^17^ Given this heterogeneity, we set up a flow‐cytometry‐based assay that could simultaneously evaluate the physical properties of EV and the expression of antigens reflecting the cell of origin. We decided to include markers of cell types involved in the pathogenesis of COVID‐19, such as platelets (CD31 and CD42b), endothelial cells (CD34 and CD31), leukocytes (CD45), pericytes (CD140b), NK cells (CD56), neural cells (CD56 and N‐cadherin) and cardiomyocytes (N‐cadherin). We did not include other excellent emerging markers that do not exhibit cell‐specific expression according to the Human Protein Atlas (https://www.proteinatlas.org).

EVs may play a complex role in viral infections because they can carry molecules that act as pathogen‐associated molecular patterns (PAMPs), triggering antiviral responses and facilitating viral propagation.^18^ In line with this, Yim and colleagues analysed 20 patients affected by a mild form of the disease and 17 healthy donors and reported an increase in CD31^+^ EV carrying subunit 1 of the SARS‐CoV2 Spike protein. Following a thorough characterization of the phenotype of small EV (<200‐nm diameter), the authors concluded that their exploratory analysis suggested the potential usefulness of serum EV in predicting disease status.^19^ A similar conclusion was drawn by Cappellano and colleagues, who compared 69 SARS‐CoV2^+^ and 62 SARS‐CoV2^−^ patients with healthy controls and defined platelet EV as a potential biomarker of COVID‐19.^20^

Our results support previous studies, and by integrating the characterization of physical and antigenic properties of EVs with clinical, blood chemistry and haematological data, we additionally demonstrate that EV have a relevant prognostic potential. Indeed, in our study, we observed that the fraction of small EV expressing either CD 31, CD 140b or CD 42b inversely correlated with patient outcomes. This result is consistent with data showing that exosomes derived from patients with mild vs. severe COVID‐19 differ in cargo composition. Specifically, mild patients have a higher abundance of exosomes positive for the SARS‐CoV2 spike protein and trigger an adaptive immune response, while severe COVID‐19‐derived exosomes are enriched in proteins associated with acute inflammatory responses.^21^ Furthermore, clinical trials that have experimented the exogenous administration of mesenchymal stem cell (MSC)‐derived exosomes to COVID‐19 patients have suggested that this intervention has a protective effect.^22^ Intriguingly, CD140b is expressed by MSC. However, we could not observe clear positivity for the exosomal marker CD63 using our flow cytometry assay.

Concerning other factors that were inversely associated with patient outcome, albuminemia and the frequency of activated monocytes expressing HLA‐DR emerged from our analysis. The association between albumin levels and COVID‐19 mortality has also been described in an independent cohort and associated with oxidative stress, neutrophil activation and thrombosis.^23^ The reduced activation of monocytes in COVID‐19 patients too is a finding that has already been described in the literature and is consistent with the immune paralysis occurring in critical patients.^7^, ^24^

Although small EVs may have a potential protective effect on the natural history of COVID‐19, a pathogenic role for EVs has also been evoked. Indeed, in a study comprising 67 patients with respiratory symptoms (34 of which were SARS‐CoV2 positives and 33 negative) and 16 healthy controls, serum‐derived EVs expressing tissue factor were the most efficient in classifying COVID‐19 patients.^25^ Importantly, the excellent prognostic role of these particles, which exert procoagulant activities in vitro, was further validated in 201 unselected consecutively enrolled patients.^26^ In line with this finding, we show that in platelet‐poor plasma samples, larger EVs expressing N‐cadherin, CD34, CD56, CD31 or CD45, of possible neuronal (or cardiac), endothelial, NK (or neuronal), endothelial and leukocyte origin, respectively, are positively associated with disease severity. Concerning the other variables positively associated with a worse outcome, we must mention age and comorbidities, identified by CCI and NT‐proBNP, as well as biomarkers of inflammation (interleukin 6) and sepsis (procalcitonin and MR, pro‐adrenomedullin), consistent with our recent data.^7^, ^8^

Finally, an intriguing result we obtained was the observed negative correlation of small EV expressing CD31, CD42b or CD140b with patient age. This result is consistent with those reported by Eitan et al.^27^ and Forest et al.^28^

In conclusion, the subpopulation analysis of circulating EV suggests that both exert protective functions and may reflect the ongoing death of specific cell types. Therefore, a comprehensive analysis that assesses at the same time EV dimensions and antigenic properties should be performed to better follow the natural history of the disease. In line with this, our complete model, which includes eight EV‐related parameters, has a good ability to discriminate patients with a worse prognosis. This study focuses on the acute systemic response to SARS‐CoV2 infection. Although the question is very important, a longitudinal study is required to verify the duration of these alterations in patients and whether they could be associated with the long‐term persistence of COVID‐19 symptoms (e.g. long‐COVID).

## AUTHOR CONTRIBUTIONS

**Federica Caponnetto:** Conceptualization (equal); data curation (equal); methodology (equal); writing – original draft (equal). **Maria De Martino:** Conceptualization (equal); formal analysis (equal); writing – original draft (supporting). **Daniele Stefanizzi:** Formal analysis (equal); investigation (equal); methodology (equal); writing – original draft (equal). **Riccardo Del Sal:** Investigation (equal); methodology (supporting). **Ivana Manini:** Formal analysis (equal). **Feras Kharrat:** Investigation (equal); methodology (supporting). **Federica D'Aurizio:** Investigation (equal); methodology (equal). **Martina Fabris:** Data curation (equal); investigation (equal); methodology (equal). **Daniela Visentini:** Investigation (equal); methodology (supporting). **Donatella Poz:** Formal analysis (equal). **Emanuela Sozio:** Investigation (equal); methodology (supporting). **Carlo Tascini:** Funding acquisition (equal); investigation (equal); methodology (equal). **Daniela Cesselli:** Data curation (equal); formal analysis (equal); investigation (equal); methodology (equal); supervision (equal). **Miriam Isola:** Formal analysis (equal); investigation (equal); methodology (equal); supervision (equal); writing – review and editing (equal). **Antonio Paolo Beltrami:** Conceptualization (lead); formal analysis (equal); supervision (lead); writing – original draft (equal); writing – review and editing (lead). **Francesco Curcio:** Formal analysis (equal); funding acquisition (equal); investigation (equal); writing – review and editing (equal).

## CONFLICT OF INTEREST STATEMENT

The authors declare no conflicts of interest.

## Supporting information


Appendix S1.

## Data Availability

The data that support the findings of this study are available from the corresponding author upon reasonable request.
