# Supplementary material for: Extracellular vesicle features are associated with COVID‐19 severity
Source: J Cell Mol Med. 2023 Nov 15;27(24):4107–17. doi: 10.1111/jcmm.17996 (PMC10746943; doi:10.1111/jcmm.17996)
Supplement: Supplementary file 1 — Appendix S1. [file JCMM-27-4107-s001.docx]

**Table S1**

|  | **Total**  **(N=98)** | **Severe**  **(N=56)** | **Critical**  **(N=42)** | **p-value** |
| --- | --- | --- | --- | --- |
| **Charlson comorbidity index, median (IQR)** | **4 (3-6)** | **3 (2-6)** | **5 (4-7)** | **0.007** |
| **Age, years, median (IQR)** | **73 (64-80)** | **71 (56-78)** | **77 (69-81)** | **0.014** |
| **Sex, n (%)**  Female  Male | 26 (26.5)  72 (73.5) | 13 (23.2)  43 (76.8) | 13 (31.0)  29 (69.0) | 0.391 |
| **Death (%)** | **26 (26.5)** | **0 (0)** | **26 (61.9)** | **<0.001** |

**Table S2**

| **Median (IQR)** | **Total**  **(N=98)** | **Severe**  **(N=56)** | **Critical**  **(N=42)** | **p-value** | **Corrected p-value** |
| --- | --- | --- | --- | --- | --- |
| C Reactive Protein (mg/L) | 85.3 (48.7-129.2) | 78.1 (38.5-122.4) | 92.4 (50.9-139.5) | 0.171 | 0.111 |
| **Proadrenomedullin (nmol/L)** | 1.16 (0.94-1.79) | 1.09 (0.82-1.43) | 1.42 (0.98-2.03) | 0.004 | **0.021** |
| **Procalcitonin (ng/mL)** | 0.15 (0.07-0.36) | 0.11 (0.06-0.23) | 0.20 (0.09-0.58) | 0.006 | **0.028** |
| **Albumin (g/L)** | 34 (31-37) | 35 (33-37) | 32.5 (30-36) | 0.014 | **0.042** |
| Total Bilirubin (mg/dL) | 0.49 (0.39-0.70) | 0.47 (0.38-0.63) | 0.59 (0.39-0.78) | 0.148 | 0.097 |
| Blood Urea Nitrogen (mg/dL) | 24 (18.5-34) | 21.5 (18-31) | 25.5 (21-37) | 0.045 | 0.062 |
| Chloride (mmol/L) | 100 (98-103) | 100 (98-103) | 101 (98-103) | 0.461 | 0.181 |
| Creatine phosphokinase (U/L) | 80 (47.5-193.5) | 72 (45-170) | 98 (49-218) | 0.571 | 0.208 |
| Creatinine (mg/L) | 1.04 (0.87-1.25) | 1 (0.83-1.26) | 1.05 (0.91-1.22) | 0.603 | 0.215 |
| Glucose (mg/dL) | 125 (103-166.5) | 126 (105-158) | 124.5 (95-167) | 0.874 | 0.229 |
| Aspartate amino transferase (U/L) | 31 (22-43) | 30 (22-39) | 37.5 (22-44) | 0.139 | 0.09 |
| Alanine amino transferase (U/L) | 25 (16-46.5) | 22 (16-45) | 34.5 (16-53) | 0.525 | 0.201 |
| Potassium (mEq/L) | 4.24 (3.84-4.56) | 4.24 (3.84-4.59) | 4.21 (3.83-4.43) | 0.933 | 0.236 |
| Lactate dehydrogenase (U/L) | 721 (541-927) | 669 (494-769) | 844 (616-992) | 0.024 | 0.056 |
| Sodium (nmol/L) | 139 (137-142) | 139 (136-142) | 139 (137-142) | 0.41 | 0.153 |
| Plasmatic osmolarity (mOsm/L) | 285 (280-291) | 284 (279-290) | 287 (281-296) | 0.109 | 0.083 |
| **N-terminal pro-B-type natriuretic peptide (pg/mL)** | 701 (223-1891) | 464 (191-1442) | 910 (499-2947) | 0.015 | **0.049** |
| **Troponin-T (µg/L)** | 16.9 (8.9-41.9) | 11.8 (7.3-27.8) | 26.8 (14.6-62.1) | 0.001 | **0.007** |
| White Blood Cells (10^3^/ µL) | 7.36 (5.39-9.78) | 7.04 (5.12-9.84) | 7.53 (5.62-9.42) | 0.46 | 0.174 |
| Red Blood Cells  (10^6^/ µL) | 4.38 (4.03-4.66) | 4.47 (4.10-4.70) | 4.36 (3.69-4.66) | 0.253 | 0.118 |
| Hemoglobin (g/dL) | 13.2 (11.9-14.2) | 13.1 (12.1-14.5) | 13.2 (11.6-14.2) | 0.512 | 0.194 |
| Hematocrit (%) | 39.6 (35.4-42.4) | 39.4 (36.7-42.1) | 40.1 (34.1-42.4) | 0.498 | 0.187 |
| Mean Corpuscular Volume (fL) | 90.9 (87.5-94.9) | 90.4 (87.5-93.2) | 91.5 (87.2-95.1) | 0.429 | 0.167 |
| Mean Corpuscular Hemoglobin (pg) | 30.6 (29.5-31.9) | 30.2 (29.5-31.9) | 31 (29.4-32.4) | 0.41 | 0.16 |
| Mean Corpuscular Hemoglobin Concentration (%) | 33.6 (33.1-34.1) | 33.6 (33-34.1) | 33.6 (33.1-34.2) | 0.956 | 0.25 |
| **Red Cell Distribution Width (%)** | 13.9 (13.2-15.5) | 13.5 (12.9-15) | 14.4 (13.6-16.4) | 0.008 | **0.035** |
| Platelets (10^3^/ µL) | 224 (159-274) | 240 (172-299) | 186 (143-264) | 0.057 | 0.076 |
| Neutrophils (%) | 85-3 (79.9-90.2) | 83.8 (79.6-89.3) | 87.3 (81.2-90-6) | 0.156 | 0.104 |
| Lymphocytes (%) | 8.3 (5.4-11.8) | 9.1 (6.1-12) | 6.8 (5-10.7) | 0.054 | 0.069 |
| Monocytes (%) | 5.3 (3.7-8.4) | 5.6 (4-8.4) | 5.3 (3-8.4) | 0.313 | 0.132 |
| Eosinophils (%) | 0 (0-0.1) | 0 (0-0.1) | 0 (0-0.1) | 0.285 | 0.125 |
| Basophils (%) | 0.2 (0.1-0.4) | 0.2 (0.1-0.4) | 0.2 (0.1-0.4) | 0.378 | 0.146 |
| D-Dimer (ng/mL) | 895 (572-1791) | 851 (497-1788) | 1007 (611.5-2120.5) | 0.338 | 0.139 |
| Prothrombin Time Rate (s) | 1.11 (1.06-1.25) | 1.11 (1.07-1.23) | 1.11 (1.06-1.32) | 0.952 | 0.243 |
| Prothrombin Time Rate international normalized ratio (s) | 1.10 (1.06-1.23) | 1.10 (1.06-1.22) | 1.10 (1.05-1.31) | 0.806 | 0.222 |
| **Interleukin 6 (pg/mL)** | 35.9 (15-101) | 27.6 (11-48) | 55.9 (25-127) | 0.001 | **0.014** |

**Table S3**

| **Median (IQR)** | **Total**  **(N=98)** | **Severe**  **(N=56)** | **Critical**  **(N=42)** | **p-value** | **Corrected p-value** |
| --- | --- | --- | --- | --- | --- |
| T Helper Lymphocytes CD4^+^ CD3^+^ (%) | 43 (34-50) | 43 (34-52) | 41.5 (32-49) | 0.354 | 0.075 |
| Cytotoxic/Suppressor T Lymphocytes CD8^+^/CD3^+^ (%) | 20 (14-28) | 21 (14-27) | 18 (13-29) | 0.444 | 0.125 |
| Natural Killer (NK) CD56^+^ CD16^+^ (%)/ CD3^-^ | 19 (11-26) | 18 (12-23) | 21 (7-28) | 0.835 | 0.25 |
| B Lymphocytes CD19^+^ (%) | 13 (8-20) | 12 (8-19) | 16 (7-20) | 0.392 | 0.1 |
| NK-like T Lymphocytes CD3^+^ /CD56 CD16^+^ (%) | 5 (1.8-9) | 5 (2.4-8.6) | 5 (1.1-9) | 0.473 | 0.175 |
| Activated T Lymphocytes CD3^+^ HLA-DR^+^ (%) | 12 (8-16) | 12 (8-16) | 11 (8-14) | 0.463 | 0.15 |
| **Activated T Helper Lymphocytes CD3^+^ CD4^+^ HLA-DR^+^ (%)** | 5 (3-7) | 5 (3-7) | 4 (3-6) | 0.278 | **0.025** |
| Activated T Cytotoxic Lymphocytes CD3^+^ CD8^+^ HLA-DR^+^ (%) | 7 (4-8) | 7 (4-8) | 6 (4-9) | 0.818 | 0.225 |
| Recent Thymic Emigrants (RTE) (%) | 16.6 (11.7-24.9) | 17.1 (10.4-25.8) | 15.8 (11.7-23.3) | 0.7066 | 0.2 |
| **Monocytes HLA-DR^+^ (%)** | 98 (90.6-99.4) | 98.3 (93.5-99.5) | 97.5 (90-99.2) | 0.297 | **0.05** |

**Table S4**

| **Median (IQR)** | **Total**  **(N=98)** | **Severe**  **(N=56)** | **Critical**  **(N=42)** | **p-value** | **Corrected p-value** |
| --- | --- | --- | --- | --- | --- |
| CD31^+^ EV (%) | 2.4 (0.9-8.2) | 2.5 (1-8) | 2.7 (0.5-10.4) | 0.677 | 0.219 |
| CD34^+^ EV (%) | 0.3 (0.1-0.5) | 0.3 (0.1-0.4) | 0.3 (0.1-0.5) | 0.736 | 0.225 |
| CD42b^+^ EV (%) | 1.1 (0.4-3.5) | 1 (0.4-3.4) | 1.8 (0.4-4.2) | 0.429 | 0.175 |
| CD45^+^ EV (%) | 0.3 (0.1-0.4) | 0.4 (0.2-0.5) | 0.2 (0.1-0.4) | 0.2452 | 0.125 |
| CD140b^+^ EV (%) | 1.9 (0.8-3.3) | 1.9 (0.8-3.3) | 2.1 (0.8-4.3) | 0.573 | 0.194 |
| CD56^+^ EV (%) | 0.6 (0.3-1.3) | 0.9 (0.3-1.6) | 0.5 (0.3-1.1) | 0.331 | 0.144 |
| N- Cadherin^+^ EV (%) | 3.1 (1.6-5.2) | 3.7 (1.6-5.2) | 2.5 (1.6-5) | 0.296 | 0.137 |
| EV/uL | 1748.8 (1044.7-3575.6) | 1672.5 (946.0-4165.7) | 2004.0 (1157.1-3355.5) | 0.625 | 0.206 |
| Small EV (%) | 19.2 (9.0-27.5) | 21.2 (9.6-32.1) | 14.5 (8.1-23.4) | 0.168 | 0.1 |
| Intermediate EV (%) | 11.2 (6.9-20.3) | 11.5 (7.0-19.7) | 10.6 (6.9-20.9) | 0.983 | 0.25 |
| Central EV (%) | 33.4 (21.1-46.7) | 29.2 (15.6-44.8) | 37.2 (25.9-49.9) | 0.077 | 0.069 |
| Large EV (%) | 22.4 (13.1-37.6) | 21.9 (13.6-35.2) | 22.4 (12.6-39.9) | 0.813 | 0.237 |
| **Small EV CD31^+^ (%)** | 5.2 (1.7-9.2) | 7.8 (2.9-13.1) | 3.1 (1.1-5.9) | 0.001 | **0.006** |
| Intermediate EV CD31^+^ (%) | 28.4 (16-41.4) | 30.1 (13.9-44.6) | 26.3 (16.9-39.2) | 0.373 | 0.169 |
| **Central EV CD31^+^ (%)** | 33.7 (24.1-46.6) | 30.3 (22.9-41.9) | 41.9 (28.8-48.0) | 0.027 | **0.044** |
| Large EV CD31^+^ (%) | 23.1 (11.4-45.5) | 23.6 (10.9-43.6) | 22.9 (14.0-47.4) | 0.6356 | 0.2125 |
| Small EV CD34^+^ (%) | 2.6 (0-6.8) | 2.7 (0-8.9) | 2.2 (0-5.6) | 0.367 | 0.156 |
| Intermediate EV CD34^+^ (%) | 4.2 (1.7-9.9) | 5.3 (0.8-11.5) | 3.8 (1.7-9.4) | 0.373 | 0.162 |
| Central EV CD34^+^ (%) | 12.8 (7.7-18.9) | 13.2 (8.2-19.0) | 11.1 (5.4-18.9) | 0.285 | 0.131 |
| **Large EV CD34^+^ (%)** | 78.3 (64.8-86.8) | 75.8 (54.3-84.4) | 80.2 (72.4-89.7) | 0.016 | **0.031** |
| **Small EV CD42b^+^ (%)** | 6.5 (2.1-10.3) | 8.2 (2.6-12.2) | 3.8 (1.8-7.8) | 0.007 | **0.019** |
| Intermediate EV CD42b^+^ (%) | 26.1 (16.9-34.4) | 27 (20.5-35.2) | 20.1 (13.3-33.3) | 0.239 | 0.119 |
| Central EV CD42b^+^ (%) | 39.1 (26.1-47.7) | 38.5 (26.1-46.2) | 39.6 (25-50.0) | 0.759 | 0.231 |
| Large EV CD42b^+^ (%) | 25.6 (14.6-40.2) | 23.6 (13.8-37.9) | 30.0 (17.2-46.8) | 0.137 | 0.081 |
| Small EV CD45^+^ (%) | 1.2 (0-2.6) | 1.5 (0-3.1) | 0.1 (0-2.3) | 0.124 | 0.075 |
| Intermediate EV CD45^+^ (%) | 3.1 (0-5.9) | 3.6 (0-7.8) | 2.3 (0.4-4.8) | 0.236 | 0.112 |
| Central EV CD45^+^ (%) | 8.8 (4.9-13.7) | 10.3 (6.1-13.8) | 8.8 (4.1-13.7) | 0.577 | 0.2 |
| **Large EV CD45^+^ (%)** | 85.4 (78.5-92.3) | 83.6 (72.1-89.1) | 89.8 (82.4-94.5) | 0.006 | **0.012** |
| **Small EV CD140b^+^ (%)** | 2.4 (1.2-4.0) | 2.8 (1.6-4.5) | 2.3 (0.6-3.1) | 0.02 | **0.037** |
| Intermediate EV CD140b^+^ (%) | 7.7 (5.2-10.4) | 8.3 (6.1-10.6) | 6.6 (4.5-10) | 0.061 | 0.056 |
| Central EV CD140b^+^ (%) | 27.9 (23.2-33.5) | 25.2 (20.5-32.7) | 28.9 (24.2-33.7) | 0.063 | 0.062 |
| Large EV CD140b^+^ (%) | 63.6 (55.9-69.6) | 62.6 (54.7-70.5) | 64.7 (57.9-69.4) | 0.533 | 0.187 |
| Small EV CD56^+^ (%) | 2.7 (1.1-6.2) | 2.9 (1.5-6.8) | 2.1 (0.8-4.5) | 0.151 | 0.087 |
| Intermediate EV CD56^+^ (%) | 9.9 (4.9-14.6) | 10.5 (4.5-15.1) | 9.5 (4.9-14.5) | 0.821 | 0.244 |
| Central EV CD56^+^ (%) | 24.3 (20-28.8) | 23.9 (16.5-28.4) | 25.1 (21.3-29.1) | 0.162 | 0.094 |
| **Large EV CD56^+^ (%)** | 60.2 (53.1-67.8) | 59.5 (46.5-66.9) | 64.8 (55.7-70) | 0.04 | **0.05** |
| Small EV N- Cadherin^+^ (%) | 6.3 (2.7-12.2) | 5.5 (2.3-11.6) | 7.1 (4.4-13.1) | 0.349 | 0.15 |
| Intermediate EV N- Cadherin^+^ (%) | 5.3 (3.4-9.4) | 4.6 (3-9.5) | 6.0 (3.7-8.7) | 0.214 | 0.106 |
| **Central EV N- Cadherin^+^ (%)** | 17.2 (11-25.3) | 15.0 (10.4-22.8) | 20.8 (13.4-28.2) | 0.016 | **0.025** |
| Large EV N- Cadherin^+^ (%) | 65.6 (47.3-79.3) | 71.1 (44.9-82.1) | 65.0 (47.3-77.3) | 0.477 | 0.181 |

**Supplementary Figure 1**

Supplementary Figure 1: Evaluation of CD63 expression in COVID-19 patient plasma (A-C) and isolated exosomes (D-F).

**Supplementary Figure 2**


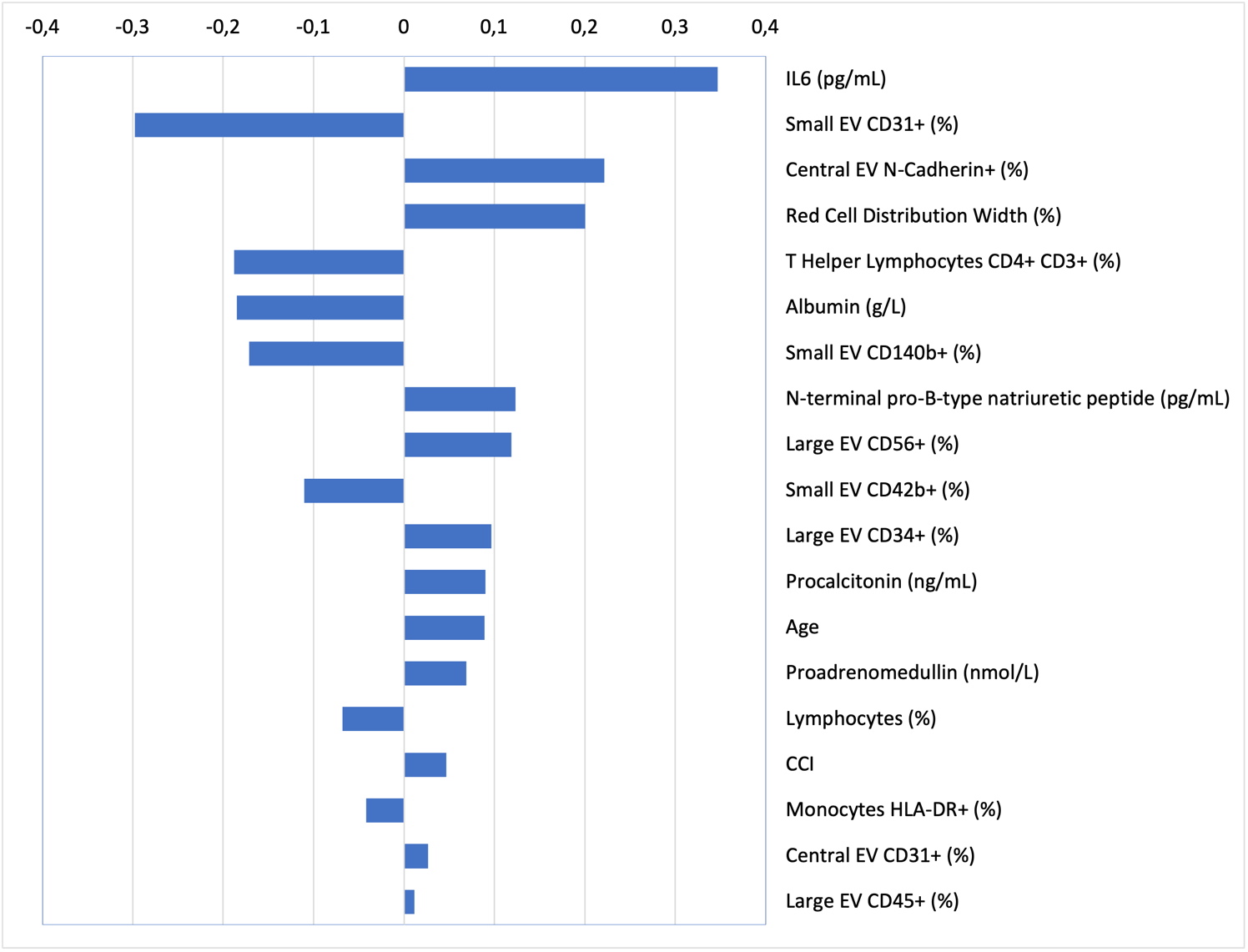


Supplementary Figure 2: Results of the elastic net logistic regression model comprising all significant variables and possible relevant confounders.
